# Detection of tobacco seedlings in plastic-mulched fields using vegetation indices and YOLOv11

**DOI:** 10.3389/fpls.2026.1951012

**Published:** 2026-09-08

**Authors:** Long Zhao, Hui Wang, Dongling Wu, Jianjun Liu, Zhe Li, Yi Shi

**Affiliations:** 1 College of Horticulture and Plant Protection, Henan University of Science and Technology, Luoyang, China; 2 Henan Province Tobacco Company, Luoyang Company, Luoyang, China; 3 Henan Province Tobacco Company, Zhengzhou, China; 4 College of Agricultural Engineering, Jiangsu University, Zhenjiang, China; 5 College of Agricultural Equipment Engineering, Henan University of Science and Technology, Luoyang, China

**Keywords:** model improvement, plastic-mulched tobacco fields, tobacco seedling detection, vegetation indices, YOLOv11

## Abstract

Missed transplants, lodged seedlings, and weak seedlings may occur during mechanized transplanting or shortly afterward, making timely post-transplant inspection essential. Plastic mulching is widely used after tobacco transplanting to improve early growth conditions. However, occlusion by the mulch film and reflections from the film surface make tobacco seedling detection more challenging in plastic-mulched tobacco fields. In this study, multispectral images of plastic-mulched tobacco fields were collected using an unmanned aerial vehicle (UAV). The normalized difference vegetation index (NDVI), green normalized difference vegetation index (GNDVI), and optimized soil-adjusted vegetation index (OSAVI) were calculated and assigned to the RGB channels in different combinations, yielding nine candidate input configurations, including six multi-index channel assignments and three single-index configurations. Screening results showed that the GNDVI-OSAVI-NDVI channel configuration enabled YOLOv11 to achieve the best overall balance of detection performance, with the highest precision, F1 score, and mAP@0.5 among the nine input configurations. Based on this optimal input strategy, depthwise separable convolution (DWSConv) and parallelized patch-aware attention (PPA) were incorporated, and the resulting model was referred to as YOLOv11-PD. Ablation experiments showed that PPA improved the main detection metrics, whereas DWSConv reduced the number of parameters and inference latency. YOLOv11-PD achieved a favorable balance between detection accuracy and computational efficiency, obtaining the highest recall, F1 score, and mAP@0.5 among all evaluated models, with values of 83.9%, 83.7%, and 0.876, respectively, while maintaining a precision of 83.5%. These results provide technical support for early-stage tobacco seedling detection under plastic mulching, evaluation of transplanting quality, and decisions on replanting missing seedlings.

## 1 Introduction

Tobacco transplanters have been increasingly adopted in mechanized tobacco production to reduce reliance on manual transplanting. Modern transplanters can improve labor efficiency and ensure relatively uniform plant spacing and transplanting depth through appropriate machine configuration and operation (Aytem et al., 2025). However, field operations may be affected by uneven land leveling, sticky and heavy soils, and variations in seedling quality, resulting in transplanting problems such as missed transplants, missing seedlings, and lodged seedlings (Li et al., 2025). If these problems are not detected and identified promptly after transplanting, the optimal window for replanting and subsequent management may be missed. Manual field inspection has limited coverage, low efficiency, and strong subjectivity, making it difficult to meet the requirements of precision management in large-scale cultivation (Vong et al., 2021, 2022). Therefore, a rapid and objective method for monitoring seedling conditions after transplanting is needed to support the assessment of transplanting quality and decisions on replanting.

To enable rapid inspection of seedling conditions after transplanting across large fields, unmanned aerial vehicles (UAVs) have been used to acquire high-resolution field images. Their advantages in rapid image acquisition, wide-area coverage, and consistent spatial resolution facilitate subsequent detection of tobacco seedlings (Liu et al., 2022; Pathak et al., 2022). Based on this capability, object detection models based on deep learning have been widely applied to crop detection in agricultural images. Among these models, the You Only Look Once (YOLO) series has been extensively used for crop detection and seedling monitoring because of its high detection efficiency (Badgujar et al., 2024). For monitoring transplanting operations, previous studies have combined YOLO-series object detection models, UAV images, and multi-object tracking to count missing seedlings in fields, identify transplanting status, and evaluate operation quality, providing a technical basis for monitoring transplanting quality (Cui et al., 2023; Wang et al., 2025; Wu et al., 2025). For tobacco, He et al. (2024) developed a YOLOv8s-EFF model based on high-resolution RGB images acquired by UAVs to detect tobacco plants at the rosette stage and further extract agronomic information, including tobacco field area, row spacing, and plant spacing. Su et al. (2025) evaluated the missed-transplanting rate for tobacco transplanters using UAV images and an improved YOLOv5s model. Their method identified planting holes in plastic mulch films with and without seedlings and then used target tracking and line-crossing counting to obtain dynamic statistics. These studies indicate that UAV images combined with YOLO-series models can effectively support the information extraction of tobacco planting and the evaluation of transplanting quality.

Previous studies have demonstrated the feasibility of detecting or segmenting tobacco plants and seedlings from RGB images acquired by UAVs. However, these applications generally rely on sufficiently visible above-ground plant features to distinguish tobacco targets from complex field backgrounds (Sun et al., 2020; Li et al., 2024a). This visibility requirement is easier to satisfy at the rosette stage, approximately 1 month after transplanting, when the tobacco canopy is more developed, and plant features are more clearly captured in RGB images (He et al., 2024). However, this timing is relatively late for seedling inspection and replanting in tobacco production, which reduces the practical value of these results for timely replanting and correction of transplanting problems. Conducting detection 1–2 weeks after transplanting enables the identification of missing seedlings and abnormal transplanting outcomes, thereby supporting early corrective measures and subsequent management decisions (Li et al., 2023; Zhao et al., 2026b). At this stage, however, tobacco seedlings remain small, and some seedlings may still be covered by mulch films. Consequently, RGB images are affected by occlusion from mulch films, spectral reflection from mulch films, and interference from their texture patterns. These factors make it difficult to capture plant features and pose challenges for detection methods during this early period (Li et al., 2024a; Zhao et al., 2026a).

When RGB images cannot reliably capture features of tobacco seedlings in plastic-mulched fields after transplanting, multispectral images can provide additional spectral information from additional bands, such as the near-infrared band. Vegetation-index layers derived from these bands can highlight vegetation signals in seedling areas, enhance contrast between seedlings and backgrounds such as mulch films, soil, and planting holes, and provide more discriminative features for models used to detect tobacco seedlings (Nitu et al., 2025; Yan et al., 2025; Zhang et al., 2025). Zhao et al. (2026a) analyzed UAV-acquired spectral data and demonstrated spectral separability among plastic mulch films, bare soil, and crop canopies. Li et al. (2024b) calculated multiple vegetation indices from multispectral images and used them as feature variables to estimate emergence rates of cotton seedlings, showing that the combined use of multiple vegetation indices reduced estimation errors. For tobacco crops, Lin et al. (2023) constructed different combinations of multispectral bands as inputs for YOLOv8 and found that the combination of red, green, and near-infrared bands outperformed RGB images, indicating that band-combination strategies can affect detection performance for tobacco targets. Chen et al. (2026) used an NDVI layer as the input for a YOLO model and incorporated strategies for multiscale feature fusion and small-object localization. The improved model achieved an APtiny of 54.6%, suggesting that vegetation-index layers can provide effective complementary features for detecting small targets such as tobacco seedlings at the early post-transplant stage. Although multispectral information and vegetation-index layers have shown value in crop recognition and seedling monitoring, strategies for mapping multiple vegetation indices into input channels and their effects on detection performance remain largely unexplored for tobacco seedlings in plastic-mulched fields during the early post-transplant stage.

To investigate vegetation-index input construction and efficient model design for rapid field inspection at the early stage after transplanting in plastic-mulched fields, this study established an overall workflow comprising UAV multispectral image acquisition and preprocessing, vegetation-index calculation and channel-mapping screening, YOLOv11-based model training and structural modification, and model performance evaluation. The specific objectives were to: (i) acquire and preprocess UAV multispectral images of tobacco seedlings at the early post-transplant stage and calculate the normalized difference vegetation index (NDVI), green normalized difference vegetation index (GNDVI), and optimized soil-adjusted vegetation index (OSAVI) as the basis for subsequent input construction; (ii) construct and compare different three-channel vegetation-index mapping schemes using the baseline YOLOv11 model and identify an appropriate input configuration for detecting normal tobacco seedlings and abnormal transplanting outcomes; and (iii) introduce depthwise separable convolution (DWSConv) and parallelized patch-aware attention (PPA) into YOLOv11 based on the selected input configuration, and evaluate the contributions of the modified modules and the combined model in terms of detection performance and computational efficiency. This study aims to provide technical support for assessing transplanting quality and replanting decisions and to offer a reference for applying multispectral information to similar tasks in detecting seedlings in plastic-mulched crops.

## 2 Materials and methods

### 2.1 Study area and data acquisition

#### 2.1.1 Study area

**Figure 1:**
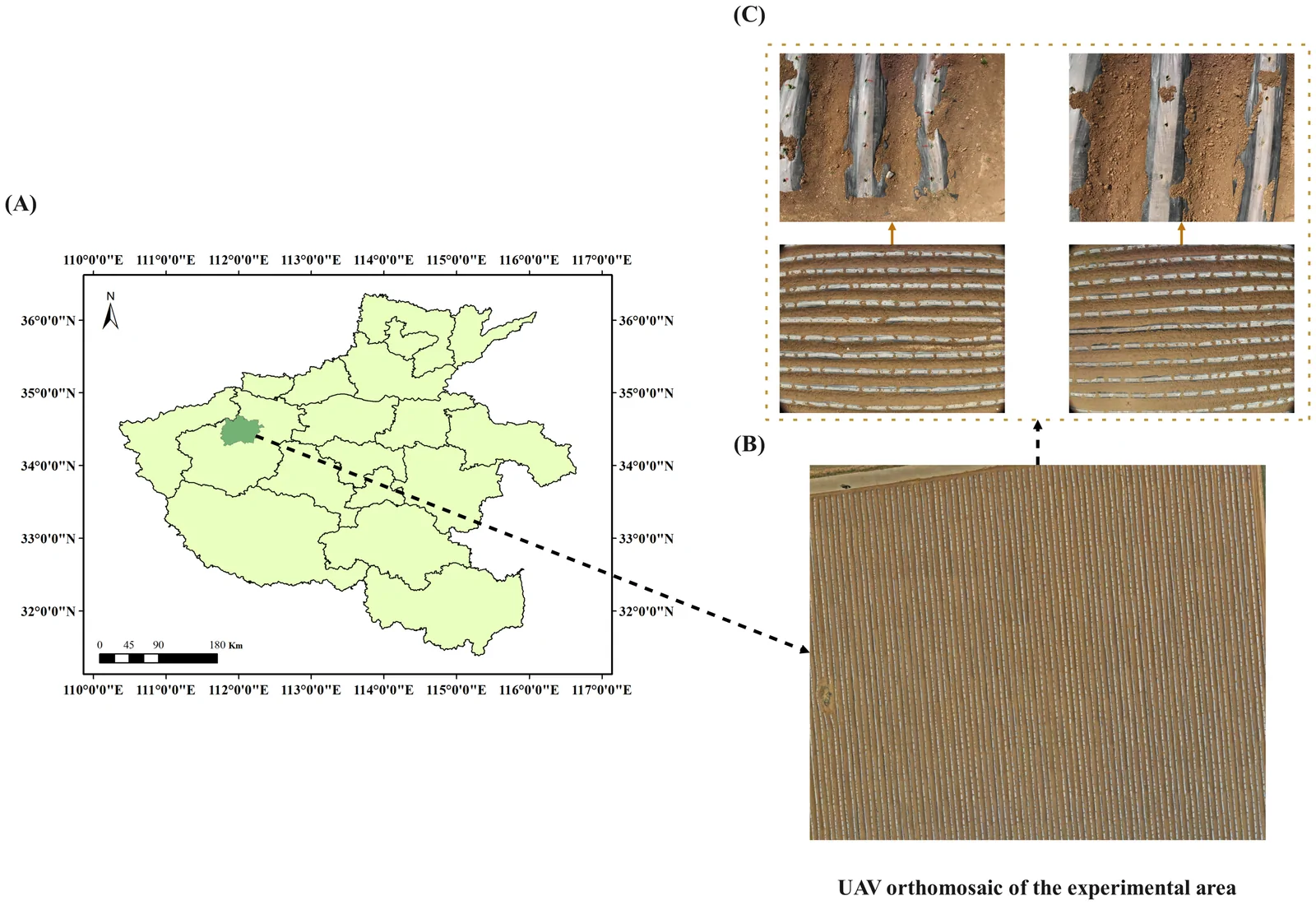
Overview of the experimental area and field conditions. **(A)** Geographic location of the study area; **(B)** UAV orthomosaic showing the layout of the experimental field, used for visualization only; **(C)** UAV images of tobacco seedlings at different spatial scales.

This study was conducted at the Luofugou experimental field in Sanxiang Town, Yiyang County, Luoyang City, Henan Province, China (34°28′5.79″ N, 111°47′46.00″ E). **Figure 1** shows the geographic location, UAV orthomosaic, and representative field views of the experimental area. The experimental site is located at an elevation of approximately 401 m, with flat terrain and yellow-brown soil. The area has a warm-temperate, semi-humid continental monsoon climate, with an annual mean temperature of 14.7 °C, a frost-free period of approximately 218 days, and an annual mean precipitation of 690.0 mm, which is concentrated from June to September. A core demonstration plot of approximately 1 ha within the experimental field was selected. The field followed a tobacco-wheat rotation, and the tobacco cultivar used was NC89.

#### 2.1.2 Multispectral data acquisition

All UAV surveys in this study were conducted at approximately 11:00 a.m. local time to minimize the effects of variations in solar elevation and shadows on multispectral image quality. UAV surveys were performed every 4 days from April 25, after tobacco transplanting was completed, to May 7, resulting in four image acquisition events. All flights were conducted under clear-sky conditions with sufficient illumination. The repeated surveys were intended to capture changes in seedling morphology and spectral responses during the early post-transplant growth stage.

Multispectral data were acquired using a DJI Mavic 3M equipped with a multispectral camera that captured four bands: green (560 ± 16 nm), red (650 ± 16 nm), red-edge (730 ± 16 nm), and near-infrared (860 ± 26 nm). Each survey used an independently planned flight route, and images were acquired at flight altitudes of 12, 6, and 3 m to obtain multiscale observations of tobacco seedlings. The median flight speeds at 3, 6, and 12 m were approximately 0.65, 1.00, and 0.40 m s^-^¹, respectively. The nominal overlap between adjacent images was approximately 30%, and the gimbal pitch angle was maintained at approximately −90°, with the camera optical axis approximately perpendicular to the ground. The original image size was 2592 × 1944 pixels. During image acquisition, the spectral sunlight sensor recorded incident-light information, which was stored together with the camera calibration and exposure parameters in the XMP metadata for subsequent processing. RGB images were stored in JPG format, whereas multispectral images were stored in TIFF format.

For each multispectral exposure, the four bands were grouped using the Capture UUID and processed following the standard workflow recommended for the DJI Mavic 3M. Manufacturer-provided calibration parameters stored in the XMP metadata were used for image correction and registration to the NIR reference grid, with minor residual offsets refined using edge-based ECC registration. The raw DN values were then normalized using the metadata-recorded black level, exposure and gain information, and sunlight-sensor irradiance. Because no reflectance calibration panel was used, the resulting values were treated as relative reflectance rather than absolute surface reflectance. Before calculating the vegetation indices, a simplified image-based radiometric correction was applied to the processed multispectral images to reduce residual additive offsets. Dark object subtraction (DOS) was applied separately to each spectral band (Wicaksono and Hafizt, 2018; Chakouri et al., 2020). The third percentile of the valid pixels in each image was used as a robust estimate of the dark-value offset, which was then subtracted from the relative-reflectance values of the corresponding spectral band, as shown in Equation 1:

(1) $$R_{B}(x,y)=max(I_{B}(x,y)-D_{B},0)$$

where *R_B_*(*x,y*) and *I_B_*(*x,y*) are the DOS-corrected and metadata-normalized relative-reflectance values, respectively, at pixel (*x, y*) in band *B*, and *D_B_* is the estimated dark-value offset for that band. Negative corrected values were clipped to zero.

### 2.2 Vegetation-index channel mapping

#### 2.2.1 Calculation of vegetation indices

After band registration, metadata-based radiometric normalization, and DOS correction, three vegetation indices (i.e., NDVI, GNDVI, and OSAVI) were selected as candidate indices using the green, red, red-edge, and near-infrared bands acquired by the DJI Mavic 3M. NDVI was used as a reference index because it is one of the most widely used indices for vegetation monitoring. GNDVI is calculated from the green and near-infrared bands and has been reported to be more sensitive to variations in chlorophyll concentration than NDVI. OSAVI is an optimized form of the soil-adjusted vegetation index that incorporates an adjustment term to reduce the influence of the soil background (Xue and Su, 2017; Yeom et al., 2019; Cheng et al., 2022). All three indices can be calculated directly from the available multispectral bands and are well-established indices, which facilitates reproducibility and comparison of the results. Rather than assuming that one index would outperform the others, this study used these indices as the basis for subsequent experiments on mapping vegetation indices to input channels and evaluating their effects on detection performance.

NDVI was calculated using Equation 2:

(2) $$NDVI=\frac{R_{NIR}-R_{R}}{R_{NIR}+R_{R}}$$

where *R_NIR_* and *R_R_* represent the processed relative-reflectance values of the near-infrared and red bands, respectively.

GNDVI was calculated using Equation 3:

(3) $$GNDVI=\frac{R_{NIR}-R_{G}}{R_{NIR}+R_{G}}$$

where *R_G_* represents the processed relative-reflectance values of the green band.

OSAVI was calculated using Equation 4:

(4) $$OSAVI=\frac{1.16\times \left(R_{NIR}-R_{R}\right)}{R_{NIR}+R_{R}+L}$$

where *L* is the soil-adjustment coefficient, which was set to 0.16 in this study.

Class labels were assigned based on ground-truth field records. During surveys, the condition of each mulch-film planting hole in the mulch film was recorded. During image annotation, these records were matched to the UAV images based on the row-wise distribution, the sequence of planting holes, and the positions of visible tobacco seedlings. Each predefined transplanting position was treated as the basic assessment unit and was assigned to one of two classes: normal tobacco seedlings or abnormal transplanting outcomes. Samples were labeled as normal tobacco seedlings if a surviving tobacco seedling was present in the corresponding planting hole, the planting position was appropriate, and the seedling morphology and growth condition satisfied the criteria for normal transplanting. Samples that did not satisfy these criteria were assigned to the abnormal transplanting outcome class, including missing seedlings, weak seedlings, lodged seedlings, leaf-damaged seedlings, and underdeveloped seedlings. These conditions were treated as representative manifestations of the abnormal transplanting outcome class rather than as separate detection subcategories.

A total of 1,620 valid original UAV images were obtained from the four acquisition dates. Because a single image could contain both normal tobacco seedlings and abnormal transplanting outcomes, the number of images was summarized by acquisition date, whereas the annotations were summarized by acquisition date and target class, as shown in **Table 1**. The number of valid images varied among acquisition dates because the repeated surveys were intended to capture representative field conditions rather than maintain an identical number of images at each survey. During the first survey, normal seedlings predominated and abnormal transplanting outcomes were relatively limited. As the post-transplant period progressed, some abnormal outcomes became more apparent, although normal seedlings remained the predominant class in the field.

**Table 1:** Distribution of UAV images and annotations across acquisition dates.

Acquisition date	Number of images	Normal tobacco seedling annotations	Abnormal transplanting outcome annotations	Total annotations
25 April	367	6325	621	6946
29 April	429	6859	1072	7931
3 May	506	7582	2019	9601
7 May	318	5074	1139	6213
Total	1620	25840	4851	30691

**Figure 2:**
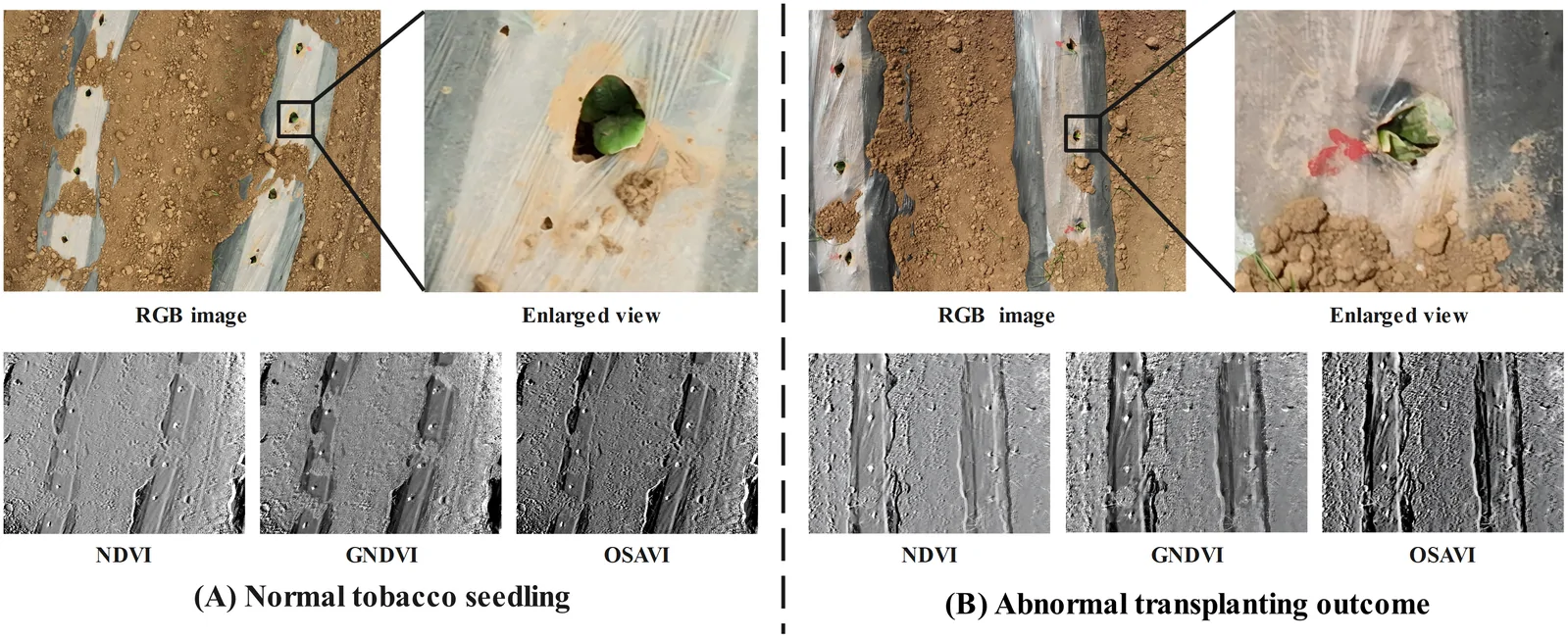
RGB and vegetation-index images of normal seedlings and abnormal transplanting outcomes. **(A)** shows normal tobacco seedling, and **(B)** shows abnormal transplanting outcome.

**Figure 2** shows representative examples of the two classes. For normal tobacco seedlings, the seedling locations were clearly identifiable within the sample regions, and relatively intact leaf structures were visible in the enlarged views. In the NDVI, GNDVI, and OSAVI images, these regions generally exhibited continuous vegetation signals. In contrast, missing seedlings produced no stable vegetation signal at the corresponding planting holes in the mulch film. Weak or underdeveloped seedlings were smaller and had incomplete leaf structures, resulting in vegetation signals with limited spatial extent, poor continuity, or low local intensity.

The original RGB images were mainly used as visual references for identifying the sequence of planting holes and the locations of visible tobacco seedlings. The final class labels were determined based on the ground-truth records, whereas the vegetation-index images were used to illustrate differences in spectral responses among different transplanting outcomes. The dataset contained two classes: normal tobacco seedlings (class 0) and abnormal transplanting outcomes (class 1). Rectangular bounding boxes were used to annotate visible seedlings or the regions corresponding to mulch-film planting holes with abnormal transplanting outcomes. The annotation results were saved in TXT format for YOLO training.

#### 2.2.2 Three-channel vegetation-index mapping and dataset construction

After band registration and radiometric correction, the NDVI, GNDVI, and OSAVI images were normalized to the same grayscale range and represented as single-channel grayscale images. To satisfy the requirement of the object detection model for three-channel input, the three vegetation-index images were spatially aligned and assigned to the red (R), green (G), and blue (B) channels. Nine channel-mapping strategies were constructed by assigning NDVI, GNDVI, and OSAVI to the three input channels either individually or in combination. The channel assignments for all input schemes are presented in **Table 2**.

**Table 2:** Assignment of vegetation indices to RGB channels under the nine mapping strategies.

No.	Mapping scheme	R channel	G channel	B channel
0	NDVI-GNDVI-OSAVI	NDVI	GNDVI	OSAVI
1	OSAVI-NDVI-GNDVI	OSAVI	NDVI	GNDVI
2	OSAVI-GNDVI-NDVI	OSAVI	GNDVI	NDVI
3	NDVI-OSAVI-GNDVI	NDVI	OSAVI	GNDVI
4	GNDVI-OSAVI-NDVI	GNDVI	OSAVI	NDVI
5	GNDVI-NDVI-OSAVI	GNDVI	NDVI	OSAVI
6	NDVI-NDVI-NDVI	NDVI	NDVI	NDVI
7	GNDVI-GNDVI-GNDVI	GNDVI	GNDVI	GNDVI
8	OSAVI-OSAVI-OSAVI	OSAVI	OSAVI	OSAVI

The three vegetation indices differed in their grayscale distribution, the spectral responses of tobacco seedlings, and local texture characteristics. In addition, different assignment orders of the three vegetation indices to the R, G, and B channels were included in the experimental design (Macaulay et al., 2026). Therefore, channel order was considered an experimental factor that could affect how the model extracts and integrates features from the three indices (Lin et al., 2023; Allu and Mesapam, 2024). All input schemes listed in **Table 2** were evaluated using the YOLOv11 baseline model under the same experimental conditions, and the best-performing strategy was selected for subsequent model development.

At the original-image level, the dataset was divided into training, validation, and test sets at a ratio of 8:1:1, containing 1,296, 162, and 162 original images, respectively. To standardize the ground coverage represented by images acquired at different flight altitudes, the 12-m and 6-m images were divided into regular image blocks of 648 × 486 pixels and 1,296 × 972 pixels, respectively, whereas the 3-m images retained their original size. The overlap between adjacent image blocks was 0%. Dataset partitioning was performed before cropping based on field spatial regions, with each original UAV image treated as the minimum grouping unit. All image blocks generated from the same original image were therefore retained within the same subset. The validation and test images were mainly selected from relatively concentrated field regions, and approximately consistent spatial ranges were maintained across acquisition dates and flight altitudes rather than performing image-level random repartitioning for each survey. After dataset partitioning, data augmentation was applied only to the training set, while the validation and test sets were kept unchanged. Before model training, all input images were resized to 640 × 640 pixels. After proportional resizing and padding to 640 × 640 pixels, the median target bounding-box size was approximately 25.7 × 24.7 pixels.

### 2.3 Detection model and architectural modifications

#### 2.3.1 Baseline YOLOv11 model

**Figure 3:**
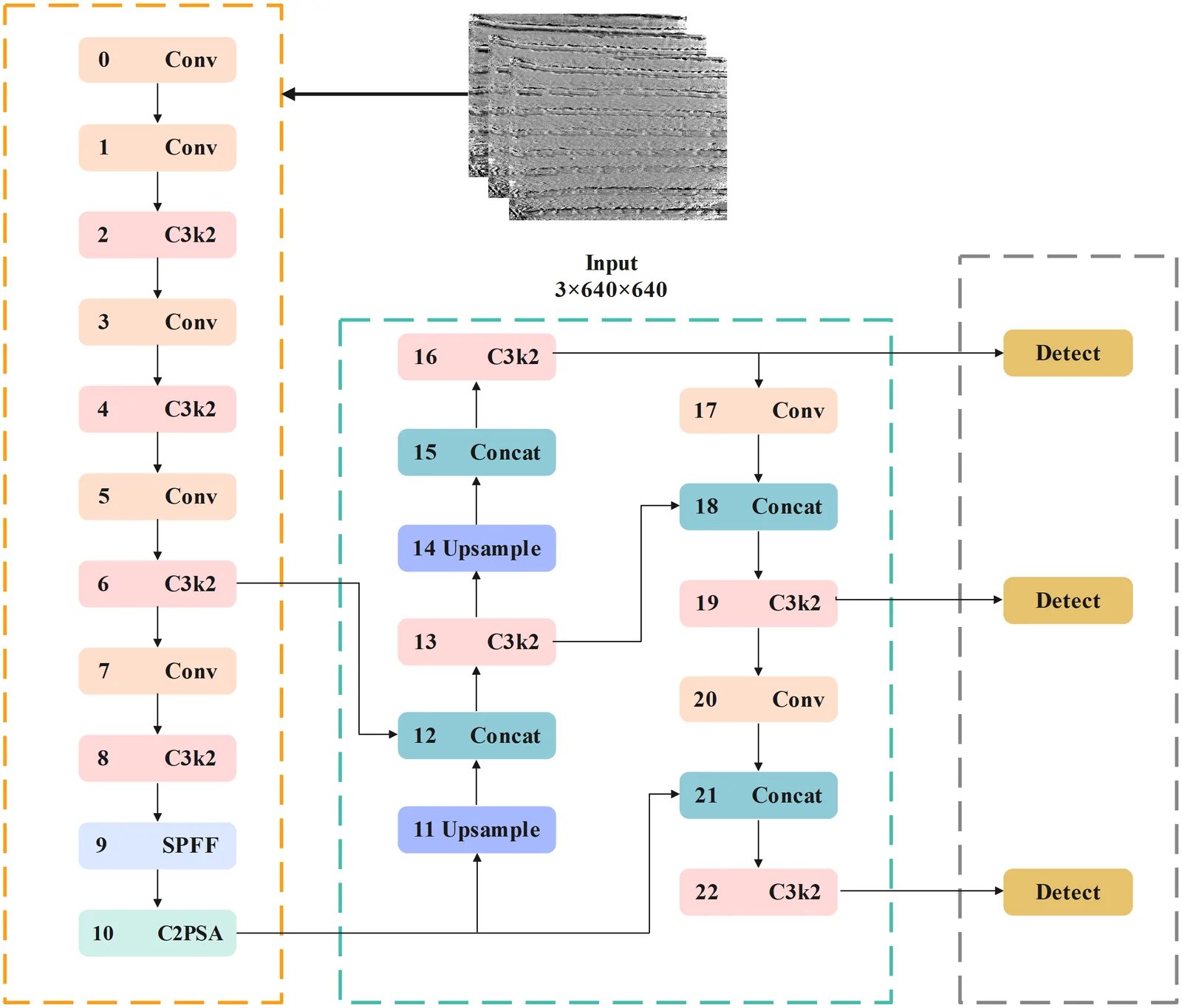
Architecture of YOLOv11.

YOLOv11 is a single-stage object detection model with a backbone for hierarchical feature extraction, a neck for multiscale feature aggregation, and a detection head for prediction (Singh et al., 2025). As shown in **Figure 3**, the inputs generated using the vegetation-index channel-mapping strategy are processed by the backbone, where Conv, C3k2, SPPF, and C2PSA modules extract image features at different levels. The neck then combines low-level spatial features with high-level semantic features through upsampling and feature concatenation. Finally, the detection head produces class predictions and bounding-box regression results at three scales.

Compared with YOLOv8, YOLOv11 incorporates C3k2 blocks in place of the earlier C2f design and adds the C2PSA module for attention-based feature refinement (Islam et al., 2026). Tobacco seedlings at the early post-transplant stage in plastic-mulched fields occupy relatively small regions in UAV images. Therefore, the multiscale detection head and attention-based feature extraction provide architectural support for detecting targets at different spatial scales. In this study, YOLOv11n was adopted as the baseline model and is hereafter referred to as YOLOv11; YOLOv5n was additionally selected as a representative mainstream lightweight baseline model for comparison. YOLOv11 was used to evaluate the nine strategies for mapping vegetation indices to channels and served as the base architecture for the subsequent structural modifications.

#### 2.3.2 Parallelized patch-aware attention module

**Figure 4:**
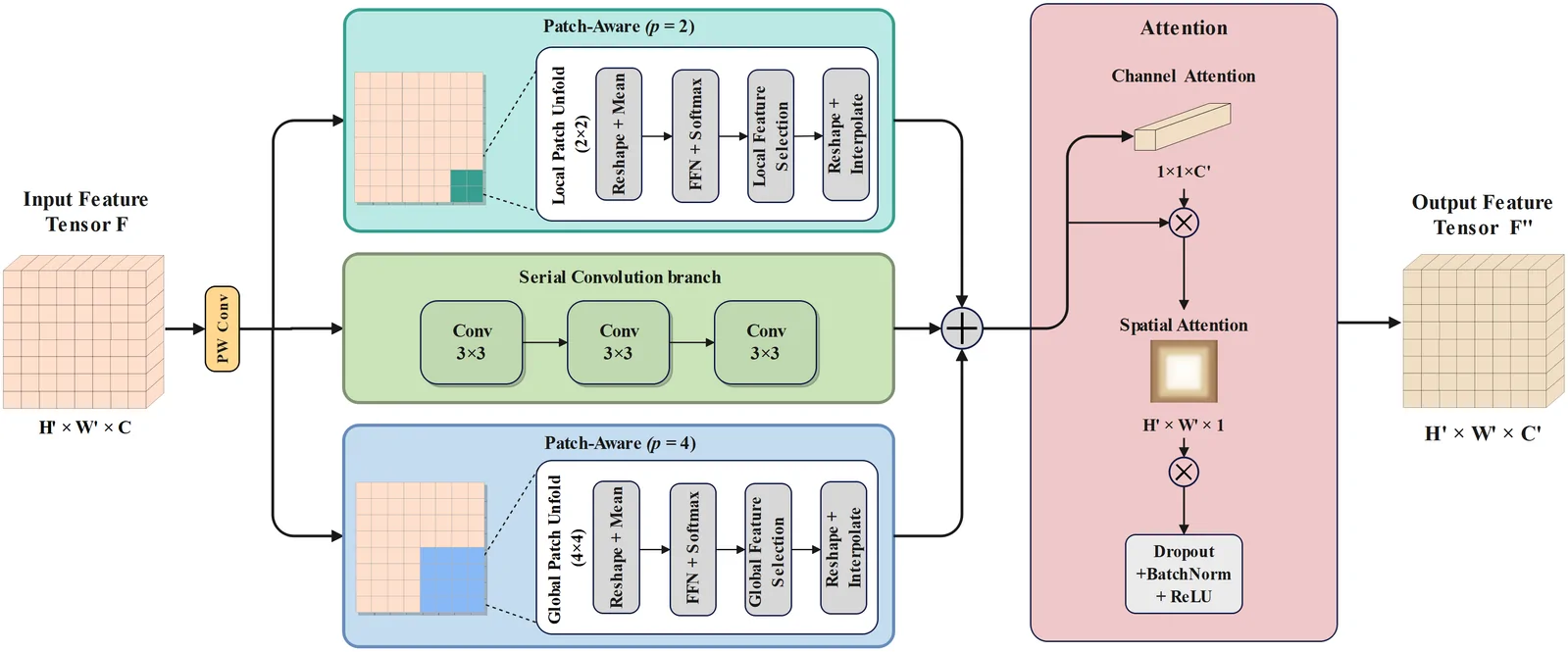
Architecture of the PPA module.

The parallelized patch-aware attention (PPA) module focuses on parallel patch-aware modeling of input features. Unlike feature extraction through sequential convolution, PPA adopts a multi-branch structure to process features in parallel at the same feature level, allowing the model to capture feature responses at different spatial scales and detail levels (Xu et al., 2024). As highlighted in HCF-Net, the module uses parallel branches—rather than a single convolution path—to obtain multiscale and hierarchical features. The architecture of the PPA module is shown in **Figure 4**.

The role of PPA in feature representation is not to modify the prediction process of the detection network, but to further organize and aggregate local information during feature extraction. After the features from the parallel branches are fused, the resulting high-level semantic features retain local structures and hierarchical feature responses while maintaining their overall abstraction capability, thereby providing richer features for subsequent fusion. In other words, PPA mainly improves the feature representation produced by the backbone rather than directly participating in classification and regression. This feature-refinement mechanism is particularly useful for preserving the weak local responses of small tobacco seedlings and reducing interference from mulch-film reflections, soil, and planting-hole textures. In the modified YOLOv11 architecture, the PPA module was inserted between the final C3k2 block and the SPPF module. The resulting model was denoted YOLOv11-PPA.

#### 2.3.3 Depthwise separable convolution module

**Figure 5:**
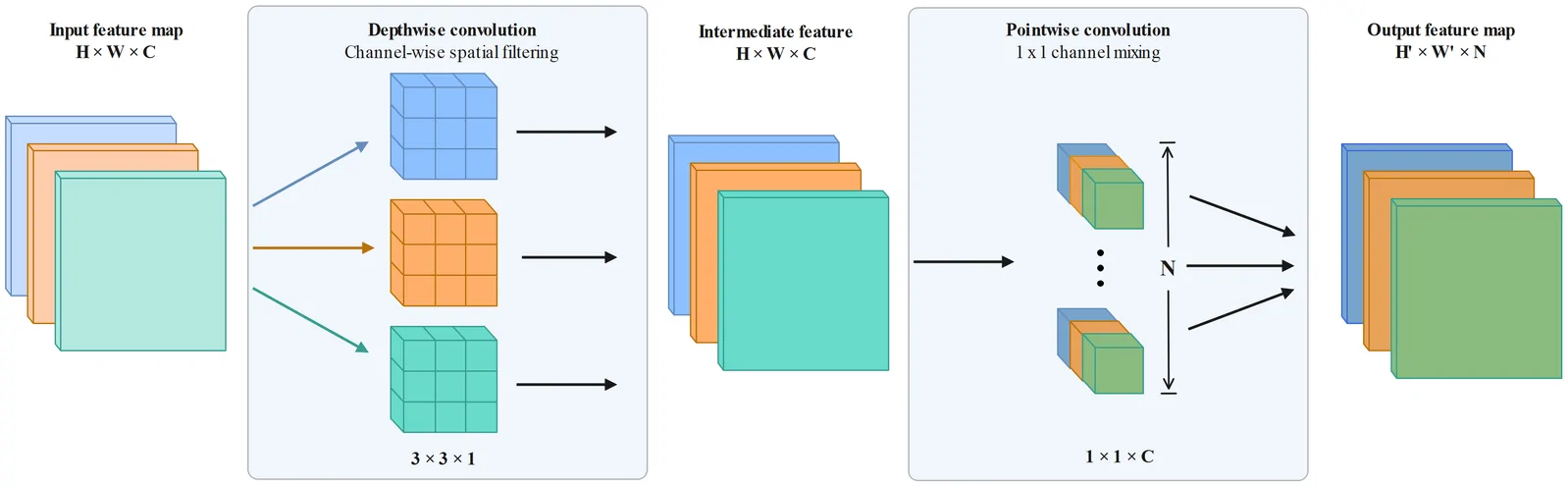
Architecture of the depthwise separable convolution module.

Depthwise separable convolution (DWSConv) is a lightweight convolutional operation that decomposes standard convolution into two steps: depthwise convolution and pointwise convolution (Chollet, 2017). Depthwise convolution applies an independent spatial kernel to each input channel to extract local spatial features. The subsequent pointwise convolution uses a 1 × 1 kernel to combine feature maps across channels. The basic structure of the DWSConv module is shown in **Figure 5**.

Compared with standard convolution, DWSConv generally requires fewer parameters and lower computational cost while maintaining spatial feature extraction and cross-channel information integration and has been applied to lightweight object-detection networks (Liu et al., 2024). In this study, DWSConv was introduced into five downsampling positions of YOLOv11 by replacing the stride-2 standard convolutional layers at layers 3, 5, and 7 of the backbone and layers 17 and 20 of the feature-fusion neck. This modification was intended to reduce the computational cost of processing high-resolution UAV images while retaining the main feature-extraction capability required for tobacco seedling detection. The resulting model was denoted YOLOv11-DWSConv. PPA and DWSConv were then integrated to construct YOLOv11-PD.

#### 2.3.4 Training configuration

YOLOv11n was used as the baseline model and was trained from random initialization without pretrained weights. SGD with a linear learning-rate schedule was used for optimization. The channel-mapping experiment was conducted for 100 epochs, whereas the subsequent ablation experiment was conducted for 200 epochs; all other training settings were kept unchanged between the two stages. HSV transformation, translation, scaling, horizontal flipping, and Mosaic were applied during training, with Mosaic disabled during the final 10 epochs. The main training settings used in the ablation experiment are summarized in **Table 3**.

**Table 3:** Main training configurations for the ablation experiment.

Parameter	Value
Batch size	8
Initial learning rate	0.01
Final learning-rate factor	0.01
Random seed	0
Validation confidence threshold	0.001
Hardware	NVIDIA RTX 3070

### 2.4 Evaluation metrics

Precision, recall, F1 score, mean average precision (*mAP*), parameter count (*Params*), and average inference latency per image (*Latency*) were used to evaluate the detection accuracy and computational efficiency of the models.

Precision represents the proportion of correctly detected objects among all objects predicted as a given class, whereas recall represents the proportion of ground-truth objects that are correctly identified. The F1 score is the harmonic mean of precision and recall. Precision, recall, and F1 score were calculated using Equations 5–7, respectively:

(5) $$Precision=\frac{TP}{TP+FP}$$

(6) $$Recall=\frac{TP}{TP+FN}$$

(7) $$F1=\frac{2\times Precision\times Recall}{Precision+Recall}$$

where *TP*, *FP*, and *FN* denote the numbers of true positives, false positives, and false negatives, respectively.

Average precision (AP) is defined as the area under the precision-recall curve for a single class, whereas mAP is calculated as the mean AP across all classes (Everingham et al., 2015). AP and mAP were calculated using Equations 8 and 9, respectively:

(8) $$AP=\int_{0}^{1}p(r)dr$$

(9) $$mAP=\frac{1}{N}\sum_{i=1}^{N}AP_{i}$$

where *p*(*r*) denotes precision at a recall level, *N* is the number of classes, and *AP_i_* is the average precision of class *i*.

Both mAP@0.5 and mAP@0.5:0.95 were used to assess overall detection performance. The former represents mAP at an intersection over union (IoU) threshold of 0.50, whereas the latter represents mAP averaged over IoU thresholds ranging from 0.50 to 0.95 in increments of 0.05 (Liu et al., 2020). The two metrics, therefore, characterize model performance under different requirements of localization accuracy.

*Params* was used to characterize model size and structural complexity. *Latency* represents the average time a model takes to process a single image. *Latency* of each model was measured under the same hardware and software environment, input size, and batch size, and averaged across all test images. Lower *Params* and *Latency* generally indicate greater computational efficiency and deployment potential.

## 3 Results and discussion

### 3.1 Selection of the strategy for mapping vegetation indices to channels

#### 3.1.1 Convergence of different channel-mapping strategies

**Figure 6:**
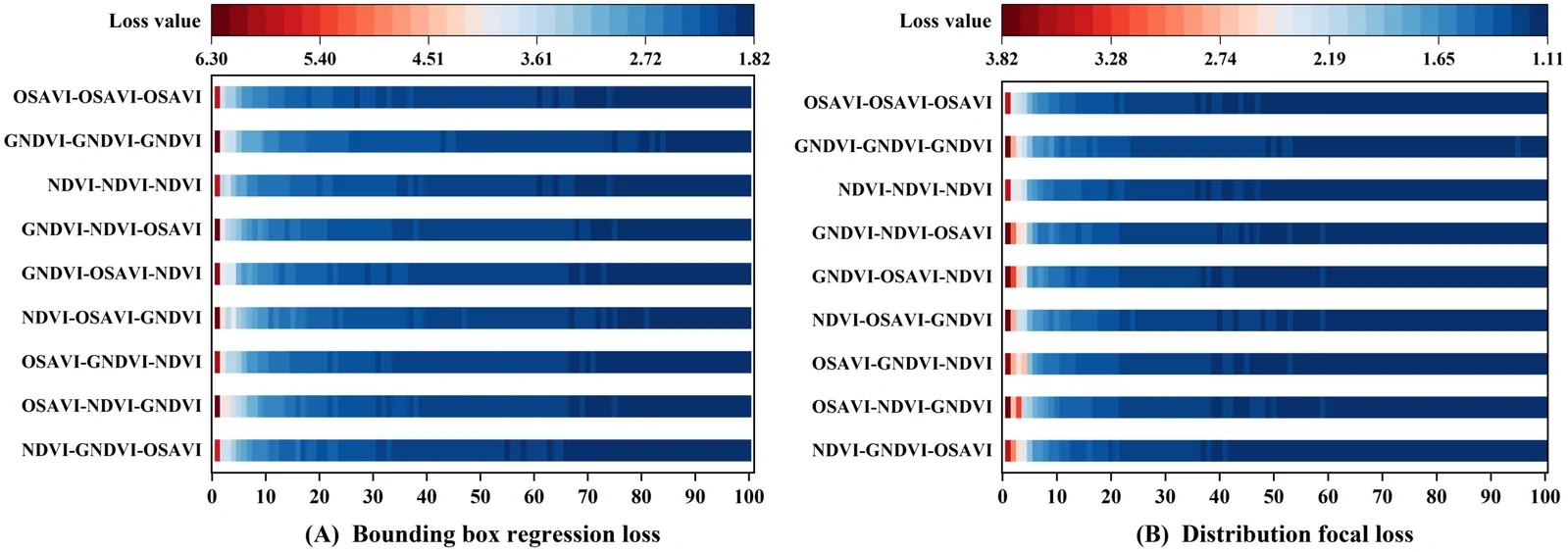
Changes in training losses for the nine vegetation-index channel mapping strategies: **(A)** bounding box regression loss and **(B)** distribution focus loss.

To evaluate the convergence behavior of the nine strategies in the baseline YOLOv11, each model was trained for 100 epochs, and the bounding-box regression loss and distribution focal loss (DFL) were recorded. As shown in **Figure 6A**, the bounding-box regression loss of all nine strategies decreased rapidly during the early training stage and then declined more gradually. This trend indicates that the localization error decreased progressively as training continued and that the models gradually achieved stable localization performance. After approximately 60 epochs, the changes in the loss curves became smaller, and the final losses stabilized at approximately 1.80. The nine strategies showed similar decreasing trends, and no sustained increase or divergence was observed. Nevertheless, differences were observed during the early training stage. The bounding-box regression loss of NDVI-OSAVI-GNDVI decreased rapidly during the early training stage and entered the low-loss range relatively early. During the later training stage, the loss values of the nine strategies gradually converged, and the differences among the nine strategies became smaller.

**Figure 6B** shows the changes in DFL. The DFL values of all nine strategies also decreased rapidly during the early training stage, indicating a reduction in fine-grained errors in bounding-box location distribution regression. After approximately 60 epochs, the curves became relatively stable and converged at approximately 1.09. Compared with the bounding-box regression loss, the differences in DFL among the nine strategies were smaller during later training. Only minor fluctuations occurred over a limited number of epochs, and no continuous increase was observed.

Overall, all nine strategies converged within 100 epochs. The differences were mainly observed in the rate of loss reduction during the early stage. NDVI-OSAVI-GNDVI entered the low-loss range relatively early for both loss functions.

#### 3.1.2 Selection of the optimal vegetation-index channel mapping strategy based on detection performance

As shown in **Table 4**, the nine strategies yielded different detection performances when evaluated with the baseline YOLOv11 model. GNDVI-OSAVI-NDVI achieved the highest precision, F1 score, and mAP@0.5, with values of 78.0%, 79.0%, and 0.828, respectively. Its recall reached 80.1%, ranking second among the nine strategies, following OSAVI-OSAVI-OSAVI at 81.3%. This finding indicates that the advantage of this assignment was reflected across multiple evaluation metrics, including overall detection performance and false-positive reduction. For mAP@0.5, GNDVI-OSAVI-NDVI improved by 3.76% compared with the second-ranked OSAVI-GNDVI-NDVI and by 11.14% compared with the lowest-performing GNDVI-GNDVI-GNDVI. These results indicate that the assignment order of vegetation indices to RGB channels influenced how the model utilized vegetation-index information.

**Table 4:** Detection performance of the nine vegetation-index channel mapping strategies.

No.	Input scheme	Precision (%)	Recall (%)	F1 score (%)	mAP@0.5
0	NDVI-GNDVI-OSAVI	74.7%	72.9%	73.8%	0.767
1	OSAVI-NDVI-GNDVI	74.3%	76.2%	75.2%	0.764
2	OSAVI-GNDVI-NDVI	75.5%	76.8%	76.1%	0.798
3	NDVI-OSAVI-GNDVI	72.4%	79.6%	75.9%	0.765
4	GNDVI-OSAVI-NDVI	78.0%	80.1%	79.0%	0.828
5	GNDVI-NDVI-OSAVI	73.2%	73.6%	73.4%	0.789
6	NDVI-NDVI-NDVI	77.4%	75.0%	76.2%	0.793
7	GNDVI-GNDVI-GNDVI	69.6%	70.0%	69.8%	0.745
8	OSAVI-OSAVI-OSAVI	69.3%	81.3%	74.8%	0.785

The main advantage of GNDVI-OSAVI-NDVI was its balance between precision and recall. Its recall reached 80.1%, which was only 1.2 percentage points lower than that of OSAVI-OSAVI-OSAVI (81.3%). However, its precision was 78.0%, which was 8.7 percentage points higher than that of OSAVI-OSAVI-OSAVI (69.3%). Thus, although OSAVI-OSAVI-OSAVI achieved a slightly higher recall, its lower precision indicated weaker false-positive reduction, which limited the corresponding improvements in F1 score and mAP@0.5. GNDVI-OSAVI-NDVI maintained high recall while achieving higher precision, resulting in an F1 score of 79.0%. Considering that the practical objective of this study is the rapid identification of tobacco seedlings and locations with abnormal transplanting outcomes for field inspection, rather than highly precise bounding-box localization, the subsequent model evaluation placed greater emphasis on mAP@0.5, recall, and inference efficiency.

### 3.2 Ablation study and performance analysis of the modified modules

**Table 5** presents the detection results of different model variants under the same input conditions. YOLOv5n achieved a precision of 72.4%, a recall of 72.1%, an F1 score of 72.2%, an mAP@0.5 of 0.783, and an mAP@0.5:0.95 of 0.268. Taking the original YOLOv11 model as the baseline, the effects of DWSConv, PPA, and their combination on detection performance were evaluated. The baseline YOLOv11 model achieved a precision of 83.0%, a recall of 79.1%, an mAP@0.5 of 0.843, and an mAP@0.5:0.95 of 0.362. Compared with YOLOv11, YOLOv5n showed decreases of 10.6, 7.0, and 8.8 percentage points in precision, recall, and F1 score, respectively, while its mAP@0.5 and mAP@0.5:0.95 were lower by 0.060 and 0.094. Although YOLOv5n had a lower inference latency, its detection performance was relatively limited in the extremely early-stage, low-contrast tobacco seedling detection scenario considered in this study. Because DWSConv and PPA were both developed on the YOLOv11n framework, the subsequent ablation analysis focuses on the YOLOv11 variants to evaluate the contribution of each module under a common base architecture.

**Table 5:** Comparison and ablation results of YOLOv5n and YOLOv11 variants in detection and deployment metrics.

Model	Precision (%)	Recall (%)	F1 score (%)	mAP@0.5	mAP@0.5:0.95	Params (M)	Latency (ms/img)
YOLOv11	83.0%	79.1%	81.0%	0.843	0.362	2.590	16.446
YOLOv11-DWSConv	77.7%	83.1%	80.3%	0.847	0.345	2.005	13.548
YOLOv11-PPA	84.2%	82.4%	83.3%	0.863	0.331	5.444	19.635
YOLOv11-PD	83.5%	83.9%	83.7%	0.876	0.342	4.176	15.084
YOLOv5	72.4%	72.1%	72.2%	0.783	0.268	2.509	3.934

After DWSConv was introduced, recall increased from 79.1% to 83.1%, corresponding to a relative increase of 5.06%, while mAP@0.5 increased slightly from 0.843 to 0.847, representing a relative increase of 0.47%. In contrast, precision decreased from 83.0% to 77.7%, with a relative decrease of 6.39%. These results indicate that DWSConv improved the coverage of tobacco seedling targets but weakened false-positive suppression. This may be because mulch-film reflections, edges of planting holes, or weakly textured background regions were more likely to be incorrectly identified as seedlings, which is consistent with the effects of background complexity and similarities between classes in object detection using optical remote sensing images (Li et al., 2020). *Params* of YOLOv11-DWSConv decreased from 2.590 M to 2.005 M, while *Latency* decreased from 16.446 to 13.548 ms. These results indicate that the main advantage of DWSConv was its ability to reduce model complexity and improve inference efficiency.

The incorporation of PPA mainly improved the model’s ability to distinguish targets. Compared with the baseline YOLOv11, precision, recall, and mAP@0.5 increased by 1.45%, 4.17%, and 2.37%, respectively. This pattern suggests that PPA improved target discrimination while avoiding the notable reduction in precision observed after incorporating DWSConv. However, these improvements came at the cost of higher computational complexity, with *Params* increasing to 5.444 M, and *Latency* rising to 19.635 ms.

When PPA and DWSConv were incorporated together, YOLOv11-PD achieved the highest mAP@0.5 among all evaluated models, reaching 0.876. Based on the values reported in **Table 5**, this represented relative increases of 3.91%, 1.51%, and 3.42% compared with the baseline YOLOv11, YOLOv11-PPA, and YOLOv11-DWSConv, respectively. YOLOv11-PD achieved a precision of 83.5%, a recall of 83.9%, and an F1 score of 83.7%, all exceeding their corresponding baseline values. The higher recall, together with the maintained precision, indicates that YOLOv11-PD reduced false negatives while preserving prediction reliability. Compared with YOLOv11-PPA, YOLOv11-PD reduced *Params* from 5.444 M to 4.176 M and decreased *Latency* from 19.635 to 15.084 ms, indicating that DWSConv partially offset the additional computational cost introduced by PPA.

Although all three modified models achieved higher mAP@0.5 than the baseline YOLOv11, their values of mAP@0.5:0.95 remained lower than the baseline value of 0.362, reaching 0.345, 0.331, and 0.342 for YOLOv11-DWSConv, YOLOv11-PPA, and YOLOv11-PD, respectively. In particular, the mAP@0.5:0.95 of YOLOv11-PD was 0.020 lower than that of the baseline model. This result indicates that the improvements provided by the modified models were mainly observed at an IoU threshold of 0.5 and did not translate into comparable gains under stricter localization requirements. One possible explanation is related to the small size of tobacco seedlings in UAV images and the characteristics of the mapped vegetation-index inputs. Because tobacco seedlings occupied only small image regions, even slight shifts in predicted bounding boxes could lead to substantial changes in IoU (Xu et al., 2022; Hua and Chen, 2025). In addition, the calculation, normalization, and channel mapping of NDVI, GNDVI, and OSAVI may enhance the contrast between seedlings and the background while reducing the contribution of fine-grained texture, edge, and morphological information present in the original imagery. These factors may partly explain why the improvements in detection performance at an IoU threshold of 0.5 were not accompanied by similar improvements under stricter localization thresholds.

**Figure 7:**
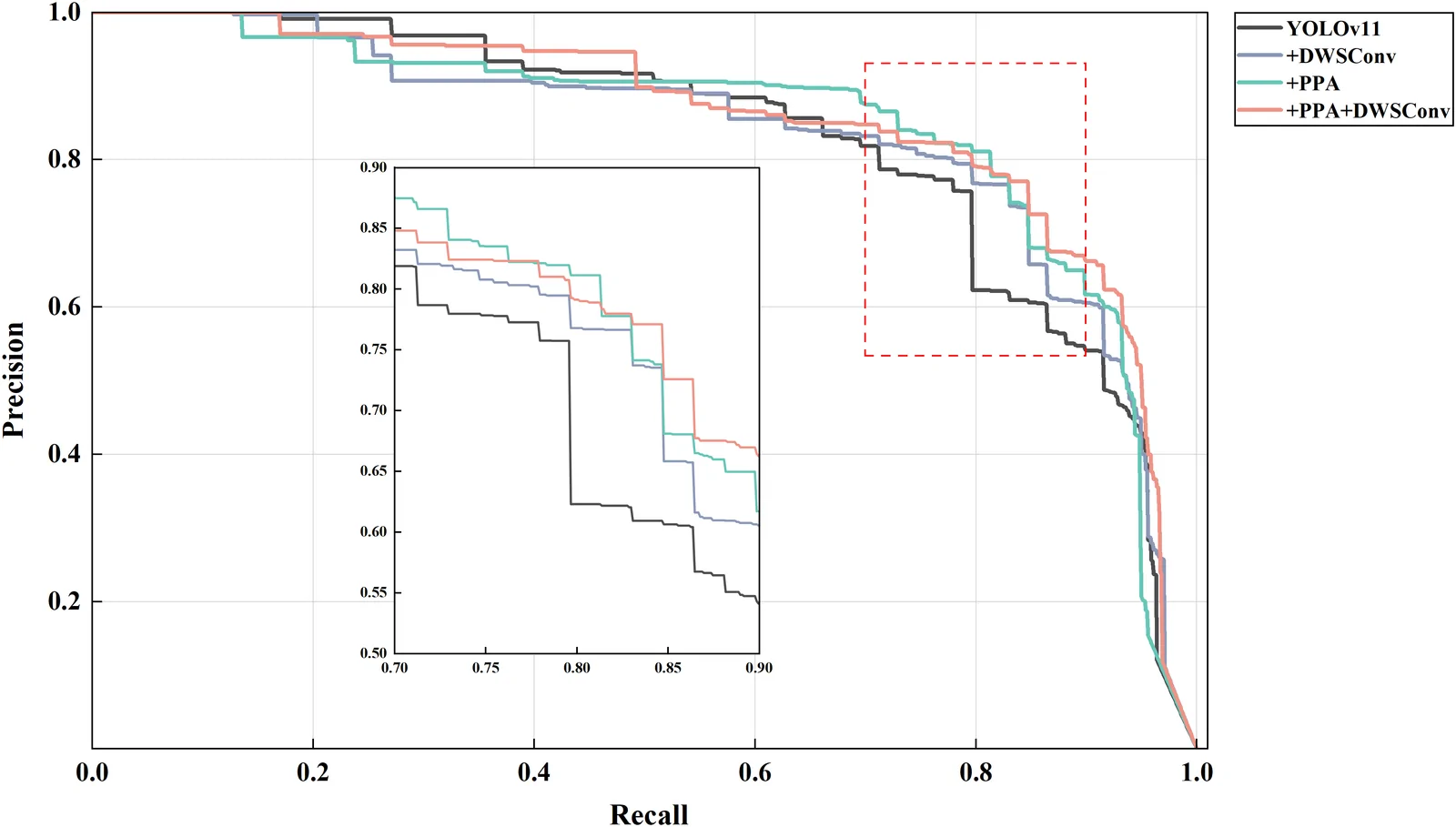
PR curves of YOLOv11 variants.

As shown in **Figure 7**, the precision-recall (PR) curves provide a perspective that varies with the confidence threshold and complements the single-value metrics. At low recall levels, all models maintained relatively high precision, and the differences among the curves were small. As recall increased, differences in false-positive control became more evident. Overall, the curves of YOLOv11-PPA and YOLOv11-PD remained higher across the medium and high recall ranges, indicating that the models incorporating PPA maintained higher precision as more targets were detected. In contrast, the curve of the baseline YOLOv11 declined earlier at higher recall levels, suggesting a greater loss of precision when the model attempted to identify more ground-truth objects.

The differences among the models were particularly evident when recall ranged from 70% to 90%. YOLOv11-PD generally maintained higher precision across this range and declined more gradually than YOLOv11 and YOLOv11-DWSConv as recall increased. This pattern suggests that the combined use of PPA and DWSConv helped maintain precision while increasing recall. YOLOv11-PPA also maintained a relatively high curve position within part of this range, suggesting that PPA contributed to maintaining precision at high recall levels. Although YOLOv11-DWSConv achieved higher recall, its precision remained lower than that of YOLOv11-PPA and YOLOv11-PD at high recall levels. Thus, DWSConv alone did not provide the same level of false-positive control as the models that incorporated PPA.

### 3.3 Visualization analysis of detection errors and feature responses

**Figure 8:**
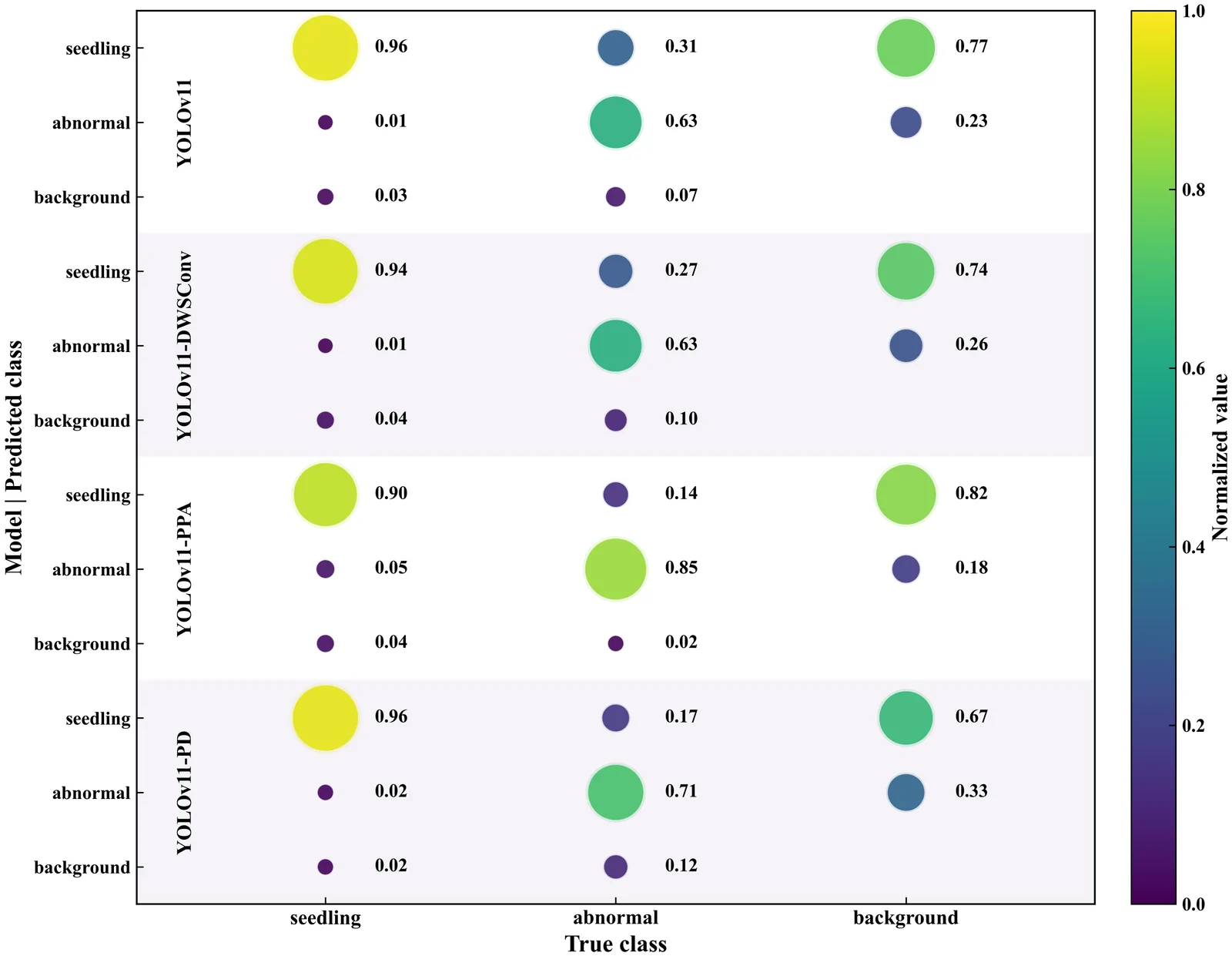
Normalized confusion matrices of the YOLOv11 variants.

The normalized confusion matrices in **Figure 8** showed that normal seedlings were not the primary source of classification errors for the models. The baseline YOLOv11 correctly classified 96% of the seedling samples, which corresponded to normal seedlings. In contrast, only 63% of the abnormal samples were correctly identified, while 31% were incorrectly classified as seedlings. These results indicate that abnormal transplanting outcomes (weak seedlings, missing seedlings, lodged seedlings, leaf-damaged seedlings, and underdeveloped seedlings) were more difficult to distinguish from normal seedlings. After DWSConv was incorporated, the proportion of abnormal samples misclassified as seedlings decreased from 31% to 27%, whereas the correct classification rate for abnormal samples remained at 63%. Thus, DWSConv only slightly reduced the confusion between the two classes and provided limited improvement in distinguishing abnormal transplanting outcomes from normal seedlings.

PPA provided a greater improvement in recognizing abnormal samples. The correct classification rate increased to 85%, while the proportion of abnormal samples misclassified as normal seedlings decreased to 14%, indicating that YOLOv11-PPA achieved better distinction between normal seedlings and abnormal transplanting outcomes. For YOLOv11-PD, 71% of the true abnormal samples were correctly classified, whereas 17% were misclassified as seedlings. Compared with the baseline model, these results represented an increase of 8 percentage points in the correct classification rate and a decrease of 14 percentage points in the confusion between abnormal samples and normal seedlings. However, YOLOv11-PD did not maintain the full improvement observed with PPA alone for abnormal-sample recognition. This suggests that the effects of PPA and DWSConv did not show a fully additive pattern, although YOLOv11-PD maintained a correct classification rate of 96% for normal seedlings.

**Figure 9:**
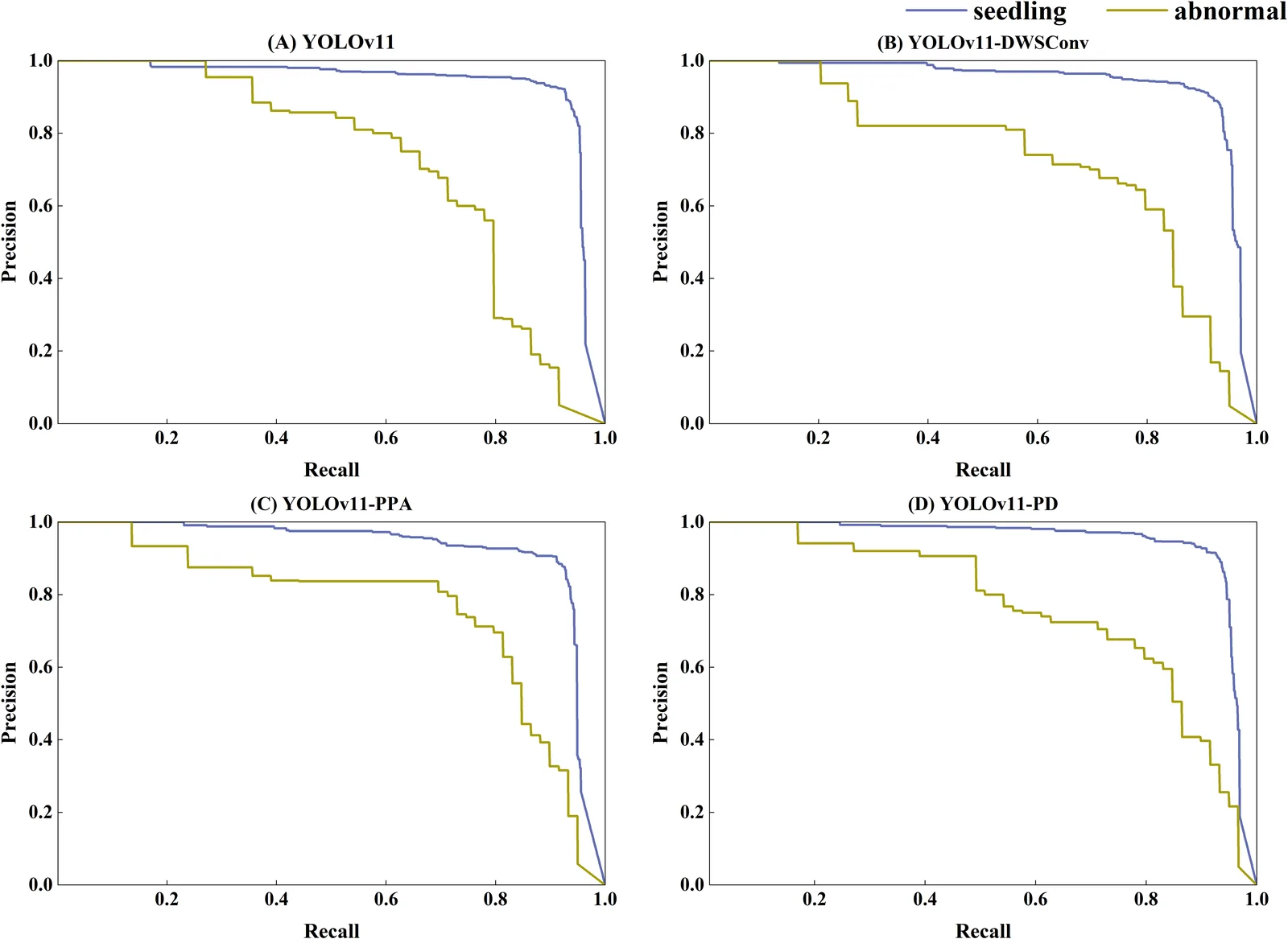
PR curves for each class of YOLOv11 variants: **(A)** YOLOv11, **(B)** YOLOv11-DWSConv, **(C)** YOLOv11-PPA, **(D)** YOLOv11-PD.

Comparison of the class-specific PR curves in **Figure 9** further showed that differences among the models were more pronounced for abnormal outcomes than for normal seedlings. Over the high-recall range from 80.0% to 95.0%, the precision for the normal seedlings remained close to 90.0% across all YOLOv11 variants. In contrast, the precision of the abnormal outcomes was consistently lower. These results indicate that predictions of normal seedlings remained reliable as recall increased, whereas abnormal transplanting outcomes were more frequently associated with false positives at high recall levels, indicating greater difficulty in distinguishing abnormal outcomes from normal seedlings.

The modified models differed primarily in how rapidly precision declined for abnormal outcomes. At a recall of 80.0%, the precision values for YOLOv11-DWSConv, YOLOv11-PPA, and YOLOv11-PD were 59.0%, 69.6%, and 62.3%, respectively, all higher than that of the baseline YOLOv11. When recall increased to 90.0%, YOLOv11-PD maintained a precision of 39.7%, compared with 32.7% for YOLOv11-PPA and 29.5% for YOLOv11-DWSConv. At a recall of 95.0%, precision for abnormal outcomes had declined to relatively low levels for YOLOv11, YOLOv11-DWSConv, and YOLOv11-PPA, whereas YOLOv11-PD still maintained a precision of 21.7%. These results indicate that precision declined more gradually for YOLOv11-PD as recall increased, allowing it to maintain higher precision in the high-recall range.

**Figure 10:**
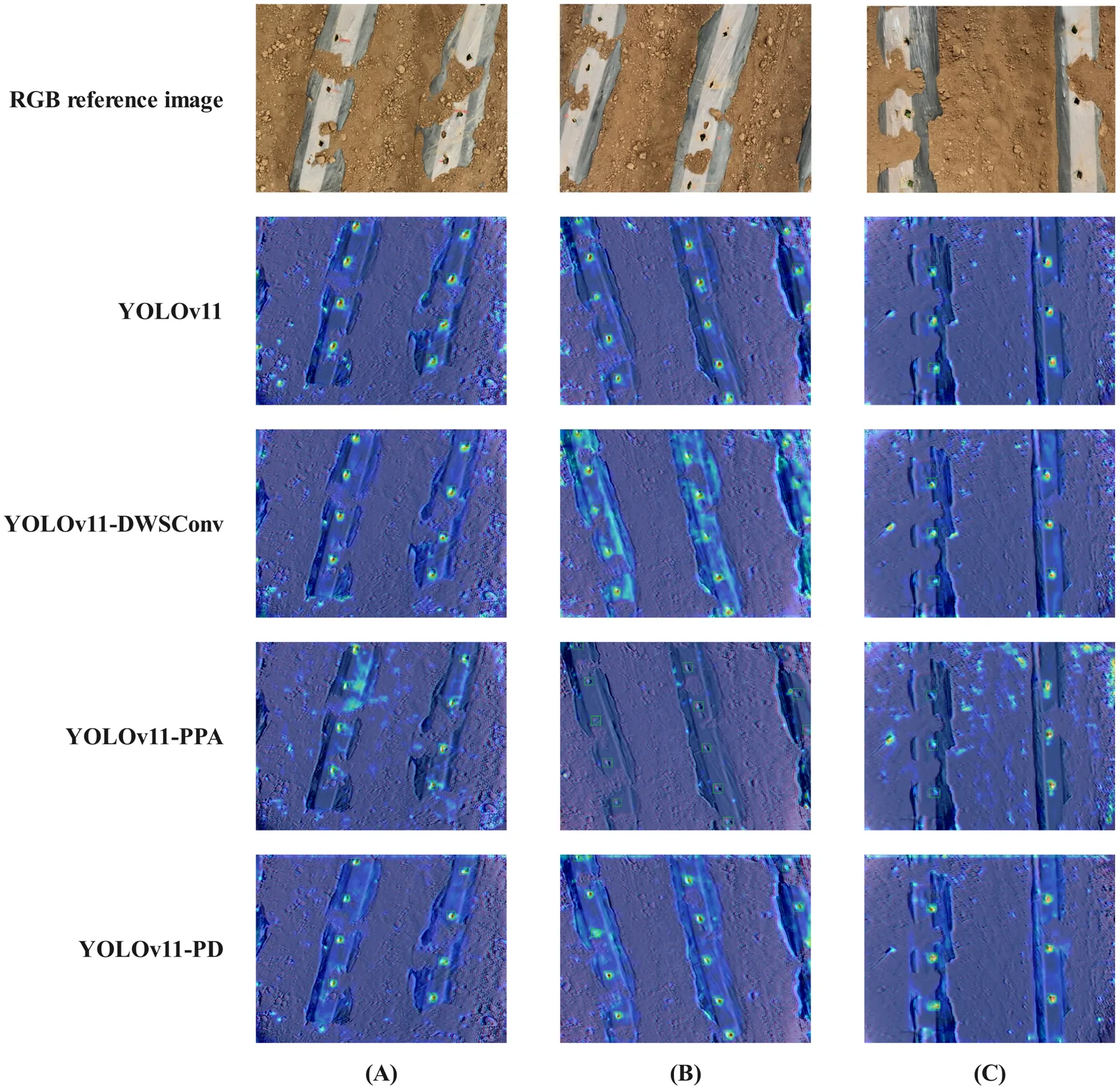
Grad-CAM visualizations of representative samples for the YOLOv11 variants. Columns **(A-C)** show three representative field samples. All heatmaps were generated using the GNDVI-OSAVI-NDVI channel configuration; the RGB images are shown only as visual references.

**Figure 10** presents the Grad-CAM heatmaps generated using the selected GNDVI-OSAVI-NDVI channel configuration to visualize the spatial distributions of feature activation. Grad-CAM generates class-discriminative localization maps by using gradients flowing into a high-level convolutional layer, thereby highlighting the image regions that contribute most to the model’s predictions (Selvaraju et al., 2020; Zhang et al., 2022). For all four models, activations were concentrated around the mulch-film planting holes, regions adjacent to the tobacco seedlings, and the surrounding background, indicating that these areas were associated with the predictions. The differences among the models were mainly reflected in two aspects. First, the spatial extent of activation along the mulch-film strips, around the edges of the planting holes, and across the mulch-film texture may indicate the influence of background features on the model predictions. Second, the extent to which the models highlighted local regions with strong vegetation-index signals reflected differences in their sensitivity to vegetation-related features.

YOLOv11 and YOLOv11-DWSConv both showed activation around the planting holes and the adjacent target areas in all three samples. However, some highly activated regions extended continuously along the mulch-film strips, around the edges of the planting holes, and across the mulch-film texture, suggesting that background features may have contributed to the model predictions. After PPA was incorporated, continuous activation over large areas of the mulch film and its texture became less evident. However, this change did not necessarily indicate that the model focused more specifically on the tobacco seedlings. In sample (B), the target seedling was almost completely covered by the mulch film, and no stable, concentrated vegetation-index signal was observed around the target. In sample (C), which contained more weeds, several non-target vegetation regions also showed pronounced activation after PPA was incorporated. This observation indicates that the attention module did not respond exclusively to tobacco seedlings but also highlighted other regions with strong vegetation-index signals. Because weeds differed from tobacco seedlings in their spatial location, size, and row-wise distribution, the stronger activation associated with weeds in the Grad-CAM heatmaps did not necessarily result in their misclassification as normal seedlings.

Compared with the other models, YOLOv11-PD maintained responses around the planting holes and the adjacent target regions in all three samples, while showing less widespread activation across the mulch-film texture. In sample (B), the regions of high activation were also more localized than those produced by YOLOv11-PPA. After PPA and DWSConv were incorporated together, the spatial distribution of activation changed, with activation remaining concentrated near the targets while continuous activation across some textured regions of the mulch film was reduced. This activation pattern was consistent with the slower decline in precision observed for YOLOv11-PD at high recall levels. However, whether these observations are causally related requires further investigation through more systematic analyses of false-positive detections and activation patterns.

## 4 Conclusion

This study investigated strategies for mapping vegetation indices to input channels and modifications to YOLOv11 for detecting tobacco seedlings at the early post-transplant stage in plastic-mulched fields. The main conclusions are as follows:

- A strategy was developed to map three vegetation indices (NDVI, GNDVI, and OSAVI) to the three RGB channels, and nine channel-mapping strategies were compared. The results showed that GNDVI–OSAVI–NDVI achieved the highest precision, F1 score, and mAP@0.5, with values of 78.0%, 79.0%, and 0.828, respectively, while maintaining a recall of 80.1%.
- Based on the optimal channel assignment for the vegetation-index inputs, DWSConv and PPA were incorporated into YOLOv11. DWSConv reduced parameter count from 2.590 M to 2.005 M and decreased average inference latency per image from 16.446 ms to 13.548 ms, although precision decreased from 83.0% to 77.7%. In contrast, PPA increased precision, recall, and mAP@0.5 to 84.2%, 82.4%, and 0.863, respectively, while increasing parameter count and average inference latency per image to 5.444 M and 19.635 ms.
- By combining PPA and DWSConv, YOLOv11-PD achieved a precision of 83.5%, a recall of 83.9%, an F1 score of 83.7%, and an mAP@0.5 of 0.876. Compared with the baseline YOLOv11, its mAP@0.5 increased by 0.033. In addition, the parameter count and average inference latency per image were both lower than those of YOLOv11-PPA. These results indicate that YOLOv11-PD achieved a favorable balance between detection performance and computational efficiency for the intended field-inspection task, although its mAP@0.5:0.95 decreased from 0.362 to 0.342, indicating a limitation under stricter localization criteria.
- Compared with the baseline YOLOv11, YOLOv11-PD improved the detection of abnormal transplanting outcomes and exhibited a slower decline in precision at high recall levels. Grad-CAM visualizations showed that the model maintained strong activation around the mulch-film planting holes and the areas adjacent to the targets, while showing less widespread continuous activation across the textured regions of the mulch film.

The proposed method can provide technical support for the assessment of transplanting quality, the identification of missing seedlings, and replanting decisions. Future research could expand the dataset with independent observations from different fields, tobacco cultivars, acquisition conditions, and post-transplant stages, and further evaluate the model’s generalizability and operational robustness through external validation.

## Data Availability

The raw data supporting the conclusions of this article will be made available by the authors, without undue reservation.
